# Latent profile analysis of depression and anxiety comorbidity in patients with acute ischemic stroke: a prospective study

**DOI:** 10.3389/fpsyt.2025.1651116

**Published:** 2025-12-08

**Authors:** Dongli Chen, Hong Zhang, Yuqi Xiu, Chunxun Xiao, Zhili Liu, Hongchun Lin, Shaoyan Zheng, Yanchun Wu

**Affiliations:** 1 Office of Nursing Research Institute, the First Affiliated Hospital of Shantou University Medical College, Shantou, Guangdong, China; 2 School of Nursing, Shantou University Medical College, Shantou, Guangdong, China; 3 Academic Affairs Office, Shantou University Medical College, Shantou, Guangdong, China

**Keywords:** acute ischemic stroke, heterogeneity, latent profile analysis, post-stroke depression, post-stroke anxiety

## Abstract

**Introduction:**

Stroke is a leading cause of mortality and disability globally, with post-stroke depression and post-stroke anxiety being common and significant complications that hinder recovery and adversely affect quality of life. Although these conditions frequently co-occur, their heterogeneity remains poorly understood. This study integrates the Health Ecology Model (HEM) and employs Latent Profile Analysis (LPA) to identify distinct psychological profiles of depression and anxiety among patients with acute ischemic stroke (AIS), as well as to investigate their multilevel determinants.

**Methods:**

Patients with AIS from a tertiary hospital in Guangdong Province, China, from January to November 2024 were included. Within one week of stroke onset, the data of sociodemographic, clinical characteristics, swallowing function, stroke severity, activities of daily living, resilience and social support were collected according to the HEM guidelines. The Patient Health Questionnaire-9 and the Generalized Anxiety Disorder-7 were used to assess the depression and anxiety symptoms of the patients three months after stroke onset. LPA was employed to identify distinct psychological profiles, and variables with a *P* < 0.1 in the univariate analysis were retained for inclusion in the subsequent multivariate analysis.

**Results:**

A total of 551 patients with AIS were included in the study, 49 were lost to follow-up or withdrew, resulting in a final analytic sample of 502 participants (91.11%). Three distinct psychological profiles were identified: no depression-anxiety (67.93%), high-risk depression-anxiety (21.12%) and major depression-anxiety (10.95%). In the multivariate analysis, the results indicated that occupation (OR = 0.61, 95% CI [0.40–0.93]), National Institutes of Health Stroke Scale (NIHSS, OR = 1.60, 95% CI [1.06–2.42]), Barthel Index (BI, OR = 1.67, 95% CI [1.27–2.19]) and hypertension (OR = 2.37, 95% CI [1.29–4.35]) were independent predictors of the high-risk depression-anxiety profile, while NIHSS (OR = 2.33, 95% CI [1.42–3.85]), BI (OR = 2.65, 95% CI [1.62–4.35]) and resilience (OR = 0.92, 95% CI [0.87–0.98]) were significantly associated with the major depression-anxiety profile.

**Conclusions:**

This study reveals significant heterogeneity in psychological distress among AIS survivors. Key predictors of post-stroke emotional comorbidity include occupation, hypertension, stroke severity, activities of daily living and low resilience. Early identification of high-risk individuals can significantly enhance screening and intervention strategies, particularly by focusing on symptoms such as anhedonia and nervousness. Future research should focus on longitudinal designs and objective biomarkers to better understand the mechanisms behind post-stroke emotional comorbidity.

## 1 Introduction

Stroke is the second leading cause of death worldwide and remains the primary cause of mortality in China, a country that accounts for approximately one-fifth of the global population (1). It is also the largest contributor to disability-adjusted life years in China (2). The high incidence, recurrence, mortality and disability rates impose a significant burden on patients, caregivers and the healthcare system (3). Stroke is classified into ischemic and hemorrhagic subtypes, with acute ischemic stroke (AIS) representing approximately 80% of cases (3). Notably, the onset of AIS is occurring at increasingly younger ages (4). By 2020, an estimated 17.8 million Chinese adults had experienced a stroke, including 3.4 million first-ever events, resulting in 2.3 million deaths that year alone (5). Although stroke-related mortality has stabilized over the past decade (6), post-stroke disability remains a major concern, with disability rates of 14.8% at three months and 14.0% at one year post-stroke (3). In addition to physical impairments, stroke frequently results in cognitive deficits (7, 8) and adversely affects mental health, with the most prominent psychological sequelae being the co-occurrence of depression and anxiety disorders (9).

Post-stroke depression (PSD) is the most prevalent neuropsychiatric complication following stroke, presenting as depressive symptoms after a cerebrovascular event (10). The incidence of PSD within two years of a stroke ranges from 11% to 41%, with the highest and most persistent risk period occurring within the first three months (11, 12). Similarly, post-stroke anxiety (PSA) is more prevalent among stroke survivors compared to the general population, though it has received relatively little attention in clinical or research contexts (13, 14). Reported PSA prevalence between three and six months after AIS ranges from 25% to 45% (15). There is a well-established association between PSD and PSA, highlighting their frequent co-occurrence and mutual reinforcement (9, 16). As China’s population continues to age and stroke incidence remains high, the combined burden of PSD and PSA is expected to increase (6). Evidence demonstrates that these comorbid conditions negatively impact recovery, quality of life and mortality risk, even raising the likelihood of recurrent stroke (17–20).

Given these significant clinical implications, it is of utmost importance to prevent and early identify PSD and PSA, especially when they occur concurrently. However, substantial heterogeneity exists in how these disorders manifest and interact in stroke survivors, leading to variable outcomes (9, 21). Traditional variable-centered approaches aggregate data across individuals and may obscure distinct subgroups (22). In contrast, person-centered methods such as Latent Profile Analysis (LPA) allow for the identification of homogeneous subpopulations based on continuous symptom severity indicators (23, 24), offering a more nuanced understanding of comorbidity patterns (25). Despite its advantages, research utilizing LPA to explore psychological symptom heterogeneity in AIS patients remains limited.

To address this gap, we applied LPA to explore latent profiles of co-occurring depression and anxiety in AIS patients. To comprehensively and systematically address the factors influencing anxiety-depression comorbidity profiles in patients with AIS, we adopted the Health Ecology Model (HEM), which incorporates personal traits, behaviors, interpersonal networks, work and living conditions and policy environment, to identify multilevel determinants of these comorbid conditions (26). The HEM underscores the multi-level interplay between environmental and personal factors and highlights the complexity inherent in their interactions. Recognizing the challenges in quantifying macro-level policy indicators (27) and acknowledging that over 95 percent of China’s population is now covered by basic medical insurance (28), our theoretical framework was constructed from four dimensions: personal traits, behaviors, interpersonal networks, work and living conditions (**Supplementary Table 1**). By integrating person-centered analysis with a comprehensive ecological perspective, this study aims to (1) characterize distinct comorbidity profiles of PSD and PSA among AIS survivors and (2) identify key predictors that differentiate these profiles.

## 2 Materials and methods

### 2.1 Study design and ethics approval statement

This prospective study adhered to the Strengthening the Reporting of Observational Studies in Epidemiology (STROBE) guidelines (**Supplementary Table 2**). Participants were recruited using convenience sampling from a tertiary general hospital in Guangdong Province, China, between January and November 2024. Ethical approval was obtained from the Institutional Review Board of the participating hospital (Approval No. SDFY B-2022-268) and all procedures complied with institutional and national ethical standards. Written informed consent was obtained from all participants prior to enrollment.

### 2.2 Participants and recruitment

#### 2.2.1 Inclusion criteria

(a) Diagnosis of AIS confirmed by cranial CT or MRI (29); (b) age ≥ 18 years; (c) conscious and able to communicate via landline or mobile phone, permitting follow-up after discharge.

#### 2.2.2 Exclusion criteria

Presence of cognitive impairment or communication disorders that would interfere with comprehension of the questionnaires.

#### 2.2.3 Drop-out criteria

Failure to establish phone contact for three consecutive days (two attempts per day) or participant-initiated withdrawal after initial contact.

### 2.3 Sample size

Sample size estimation was based on Kendall’s rule, which recommends 5~10 participants per variable for multivariate analyses (30). With 20 sociodemographic variables, Water Swallowing Test (WST), National Institutes of Health Stroke Scale (NIHSS), Barthel Index (BI), 10-item Connor–Davidson Resilience Scale (CD-RISC-10) and Social Support Rating (SSRS), a minimum of 125 to 250 participants was required. Allowing for a 10% drop-out rate, the final target sample size ranged from 139 to 278 participants. Furthermore, previous studies recommend a sample size of 300 to 1000 for LPA (31). The study ultimately included 502 participants.

### 2.4 Instrument

Key independent variables, including sociodemographic data, disease-related characteristics, laboratory indicators, stroke severity, swallowing function, activities of daily living, resilience scale and social support scale were collected within one week of disease onset. Depression and anxiety were assessed via phone follow-up at 3 months post-stroke.

#### 2.4.1 Sociodemographic and clinical characteristics

A structured questionnaire collected data on age, sex, height (HT), weight (WT), education level, smoking and drinking history, marital status, occupation and primary caregiver. Clinical data included number of previous strokes, hypertension, diabetes and swallowing function. Laboratory indicators included: (a) hematological: platelet count, neutrophils, lymphocytes; (b) nutritional: albumin; (c) lipid profile: total cholesterol (TC), low-density lipoprotein cholesterol (LDL-C), high-density lipoprotein cholesterol (HDL-C) and triglycerides (TG). Derived indices: Body Mass Index (BMI = WT/HT², kg/m²), categorized as underweight (<18.5), healthy (18.5~23.9), overweight (24.0~27.9), or obese (≥28.0) (32); Neutrophil-to-lymphocyte ratio (NLR = neutrophils/lymphocytes); Platelet-to-lymphocyte ratio (PLR = platelets/lymphocytes); Systemic immune-inflammation index (SII = neutrophils × platelets/lymphocytes); Prognostic nutritional index (PNI = albumin + 5 × lymphocytes); Atherogenic indices including Atherogenic Index of Plasma (AIP = log [TG/HDL-C]), Castelli’s-Risk-Index-I (CRI-I = TC/HDL-C), Castelli’s-Risk-Index-II (CRI-II = LDL-C/HDL-C), and Atherogenic-Coefficient (AC = [TC−HDL-C]/HDL-C).

#### 2.4.2 Swallowing function

Swallowing ability was assessed using the WST (33). The assessment team observed each participant’s response after ingesting 30 ml of water, with the following classifications: first degree – able to swallow 30 ml of water smoothly in one sitting; second degree – able to swallow 30 ml of water in two or more attempts without choking or coughing; third degree – able to swallow 30 ml of water in one attempt, but with choking; fourth degree – able to swallow 30 ml of water in small portions, but with choking; fifth degree – frequent choking and coughing, unable to swallow 30 ml of water.

#### 2.4.3 Stroke severity

NIHSS is a reliable, valid and responsive tool for measuring stroke severity (34). Total scores range from 0 to 42, with higher scores indicating more severe neurological deficits. Stroke severity was categorized as: mild (0~4), intermediate (5~9), moderate (10~14) and severe (≥15) (35, 36).

#### 2.4.4 Activities of daily living

BI evaluates functional independence in activities of daily living across 10 domains (37). Scores range from 0 to 100, categorized as: total dependency (0~20), severe dependency (21~60), moderate dependency (61~90), slight dependency (91~99) and independence (100) (38).

#### 2.4.5 Resilience

Psychological resilience was assessed using the CD-RISC-10 (39). Each item is rated on a 5-point scale (0~4), with total scores ranging from 0 to 40. Higher scores denote greater resilience.

#### 2.4.6 Social support

Social support was assessed using the SSRS, widely used in Chinese populations (40). It comprises three components: subjective support, objective support and support utilization. Given the absence of consensus on cut-off values, the total score was treated as a continuous variable in analyses (41).

#### 2.4.7 Depression

The Patient Health Questionnaire-9 (PHQ-9), a validated screening tool for PSD (42), comprises nine items scored from 0 to 3. Total scores range from 0 to 27, with higher scores reflecting greater severity of depressive symptoms.

#### 2.4.8 Anxiety

Anxiety symptoms were evaluated using the Generalized Anxiety Disorder-7 (GAD-7) scale, a seven-item instrument designed to assess anxiety frequency and severity (43). Scores range from 0 to 21.

### 2.5 Data collection and quality assurance

Trained research personnel explained the study procedures and obtained written informed consent at hospital admission. Baseline assessments, including demographics, clinical characteristics and laboratory indicators, were recorded. Swallowing function and NIHSS were evaluated by a team comprising trained researchers and clinicians. Instruct the patients to fill in the CD-RISC-10 and SSRS. Depression and anxiety assessments were conducted via telephone at 3 months (44). All data were independently entered and double-checked by two researchers using EpiData 3.1 to ensure accuracy.

### 2.6 Statistical methods

Missing data were handled using multiple imputation. To assess potential common method bias, Harman’s single-factor test was performed. Descriptive statistics were conducted using SPSS version 26.0. Categorical variables were presented as frequencies and percentages; continuous variables as means ± standard deviations (M ± SD). The Kolmogorov–Smirnov test was used to assess normality, revealing non-normal distributions for all continuous variables.

LPA was performed in R version 4.4.3 using the tidyLPA package (45). Model fit was evaluated using the Akaike Information Criterion (AIC), Bayesian Information Criterion (BIC), sample size adjusted BIC (aBIC), entropy and the Bootstrap Likelihood Ratio Test (BLRT) (46). Lower AIC, BIC and aBIC values indicated superior model fit. Entropy values ≥0.80 were considered acceptable and a significant BLRT *P* supported the inclusion of additional classes (47). Models were also required to include no latent class smaller than 5% of the sample (48).

Between-profile comparisons were conducted using the Kruskal–Wallis test for continuous variables and chi-square or Fisher’s exact test for categorical variables, as appropriate. Due to unequal class sizes, Firth’s penalized likelihood logistic regression was used for multivariable analysis (49), which reduces small-sample bias and addresses issues such as separation (50, 51). Variables significant in univariate analyses (*P* < 0.1) were screened for multicollinearity (52); no multicollinearity was detected. Final models reported odds ratios (OR) and 95% confidence intervals (CI). A *P* < 0.05 was considered statistically significant.

## 3 Results

### 3.1 Participant characteristics

**Figure 1:**
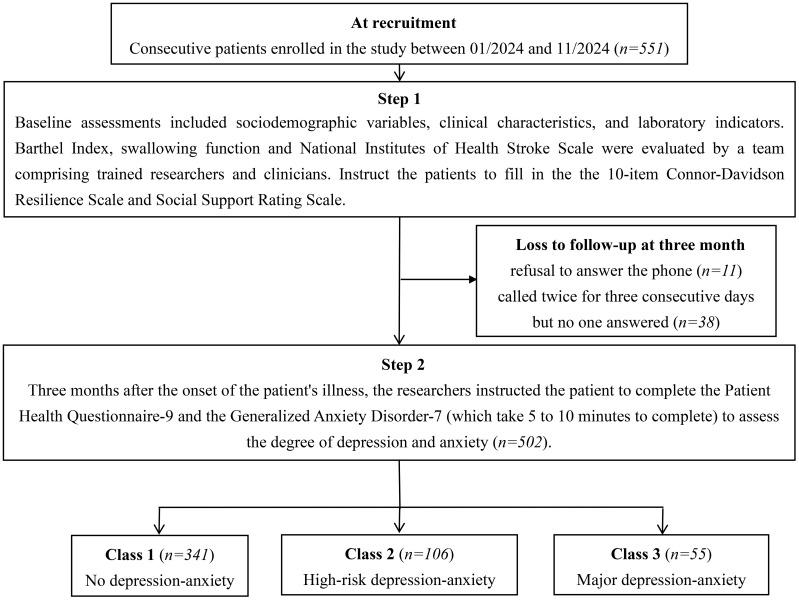
Study flowchart.

Out of the 551 individuals initially enrolled in the study, 49 were lost to follow-up or chose to discontinue participation. As a result, a total of 502 participants (91.11%) were included in the final analysis (**Figure 1**). The mean age of the participants was 67.19 years (SD = 0.48), with 65.54% being male and 97.61% married. A detailed breakdown of the demographic and clinical characteristics of the AIS patients is provided in **Table 1**.

**Table 1:** General information of patients with AIS and the differences in the potential profile groups.

Variables	All participants (N = 502) N (%) / M ± SD	Class 1 (N = 341) N (%) / M ± SD	Class 2 (N = 106) N (%) / M ± SD	Class 3 (N = 55) N (%) / M ± SD	*X*^2^/ H	*P*
Marital status, n					1.06^#^	0.731
**Married**	490 (97.61)	331 (97.07)	105 (99.06)	54 (98.18)		
**Single**	12 (2.39)	10 (2.93)	1 (0.94)	1 (1.82)		
Gender, n					3.37	0.185
**Male**	329 (65.54)	227 (66.57)	72 (67.92)	30 (54.55)		
**Female**	173 (34.46)	114 (33.43)	34 (32.08)	25 (45.45)		
Smoke, n					1.34	0.512
No	257 (51.20)	170 (49.85)	55 (51.89)	32 (58.18)		
Yes	245 (48.80)	171 (50.15)	51 (48.11)	23 (41.82)		
Drink, n					0.19	0.909
No	419 (83.47)	286 (83.87)	87 (82.08)	46 (83.64)		
Yes	83 (16.53)	55 (16.13)	19 (17.92)	9 (16.36)		
Education status, n					8.97	0.345
Illiteracy	78 (15.54)	58 (17.01)	12 (11.32)	8 (14.55)		
Primary school	226 (45.02)	149 (43.70)	53 (50.00)	24 (43.64)		
Junior high school	105 (20.92)	75 (21.99)	23 (21.70)	7 (12.73)		
Senior high school/Secondary school	72 (14.34)	44 (12.90)	15 (14.15)	13 (23.64)		
Junior college/Bachelor's degree or above	21 (4.18)	15 (4.40)	3 (2.83)	3 (5.45)		
Occupation, n					11.31^#^	0.062
Retirement	92 (18.33)	51 (14.96)	28 (26.42)	13 (23.64)		
Unemployed	326 (64.94)	226 (66.28)	65 (61.32)	35 (63.64)		
**Employed**	77 (15.34)	60 (17.60)	11 (10.38)	6 (10.91)		
**Farmers/Others**	7 (1.39)	4 (1.17)	2 (1.89)	1 (1.82)		
Primary caregiver, n					6.87^#^	0.303
**Spouse**	240 (47.81)	167 (48.97)	48 (45.28)	25 (45.45)		
**Children**	209 (41.63)	132 (38.71)	50 (47.17)	27 (49.09)		
**Oneself**	43 (8.57)	35 (10.26)	5 (4.72)	3 (5.45)		
**Porter/Others**	10 (1.99)	7 (2.05)	3 (2.83)	0 (0.00)		
Hypertension, n					17.01	<0.001
**No**	145 (28.88)	118 (34.60)	17 (16.04)	10 (18.18)		
**Yes**	357 (71.12)	223 (65.40)	89 (83.96)	45 (81.82)		
Diabetes, n					1.36	0.507
**No**	340 (67.73)	236 (69.21)	70 (66.04)	34 (61.82)		
**Yes**	162 (32.27)	105 (30.79)	36 (33.96)	21 (38.18)		
Number of Previous Strokes, n					4.85^#^	0.543
**No**	366 (72.91)	245 (71.85)	77 (72.64)	44 (80.00)		
**Once**	102 (20.32)	75 (21.99)	20 (18.87)	7 (12.73)		
**Twice**	22 (4.38)	12 (3.52)	7 (6.60)	3 (5.45)		
**Three times or more**	12 (2.39)	9 (2.64)	2 (1.89)	1 (1.82)		
BMI, n					6.96	0.325
**Underweight**	32 (6.37)	23 (6.74)	4 (3.77)	5 (9.09)		
**Healthy**	279 (55.58)	182 (53.37)	60 (56.60)	37 (67.27)		
**Overweight**	154 (30.68)	110 (32.26)	34 (32.08)	10 (18.18)		
**Obese**	37 (7.37)	26 (7.62)	8 (7.55)	3 (5.45)		
NIHSS, n					91.53^#^	<0.001
**Mild**	357 (71.12)	283 (82.99)	55 (51.89)	19 (34.55)		
**Intermediate**	98 (19.52)	47 (13.78)	33 (31.13)	18 (32.73)		
**Moderate**	41 (8.17)	8 (2.35)	18 (16.98)	15 (27.27)		
**Severe**	6 (1.20)	3 (0.88)	0 (0.00)	3 (5.45)		
WST, n					75.83^#^	<0.001
**Grade 1**	254 (50.60)	199 (58.36)	41 (38.68)	14 (25.45)		
**Grade 2**	116 (23.11)	86 (25.22)	23 (21.70)	7 (12.73)		
**Grade 3**	73 (14.54)	34 (9.97)	20 (18.87)	19 (34.55)		
**Grade 4**	16 (3.19)	11 (3.23)	2 (1.89)	3 (5.45)		
**Grade 5**	43 (8.57)	11 (3.23)	20 (18.87)	12 (21.82)		
BI, n					103.90	<0.001
**Dependence**	58 (11.55)	56 (16.42)	2 (1.89)	0 (0.00)		
**Slight dependence**	80 (15.94)	68 (19.94)	12 (11.32)	0 (0.00)		
**Moderate dependence**	111 (22.11)	92 (26.98)	15 (14.15)	4 (7.27)		
**Severe dependence**	178 (35.46)	100 (29.33)	48 (45.28)	30 (54.55)		
**Total dependence**	75 (14.94)	25 (7.33)	29 (27.36)	21 (38.18)		
Age, Years	67.19 (0.48)	66.32 (0.56)	67.98 (1.10)	71.04 (1.66)	10.57	0.005
CD-RISC-10	27.17 (0.40)	28.13 (0.44)	26.17 (0.98)	23.18 (1.45)	11.88	0.003
SSRS	39.67 (0.28)	40.14 (0.33)	39.58 (0.60)	36.95 (0.86)	12.53	0.002
PNI	49.41 (0.70)	50.23 (0.87)	46.61 (0.46)	49.69 (3.26)	19.16	<0.001
SII	873.47 (31.01)	806.13 (32.61)	1001.15 (78.18)	1044.84 (123.95)	12.50	0.002
NLR	3.87 (0.13)	3.52 (0.14)	4.62 (0.35)	4.63 (0.50)	18.59	<0.001
PLR	139.98 (3.27)	133.07 (3.70)	149.19 (7.32)	165.10 (12.16)	12.51	0.002
AIP	0.09 (0.01)	0.09 (0.02)	0.10 (0.03)	0.01 (0.03)	3.16	0.206
AC	3.82 (0.14)	3.91 (0.19)	3.79 (0.15)	3.34 (0.15)	3.45	0.178
CRI-I	4.82 (0.14)	4.91 (0.19)	4.79(0.15)	4.34 (0.15)	3.45	0.178
CRI-II	2.92 (0.05)	2.91 (0.06)	3.06(0.12)	2.73 (0.12)	2.65	0.266

^#^Fisher's exact test.

BMI, Body Mass Index; NIHSS, the National Institutes of Health Stroke Scale; WST, Water Swallowing Test; BI, Barthel Index; CD-RISC-10, the 10-item Connor-Davidson Resilience Scale; SSRS, Social Support Rating Scale; PNI, Prognostic Nutritional Index; SII, Systemic Immune-inflammation Index; NLR, Neutrophil-to-Lymphocyte Ratio; PLR, Platelet-to-Lymphocyte Ratio; AIP, Atherogenic Index of Plasma; AC, Atherogenic-Coefficient; CRI-I, Castelli's-Risk-Index-I; CRI-II, Castelli's-Risk-Index-II.

### 3.2 Tests for common method bias and multicollinearity

Given the use of self-reported data, Harman’s single-factor test was conducted through exploratory factor analysis to assess potential common method bias. Eight factors with eigenvalues greater than 1 were extracted, explaining a total of 61.93% of the variance. The first factor accounted for only 12.17% of the variance, which is well below the commonly accepted threshold of 40% (53), indicating that common method bias was not a concern.

To further validate the analysis, multicollinearity diagnostics were performed. Tolerance values ranged from 0.12 to 0.95, and the variance inflation factors (VIFs) ranged from 1.06 to 8.32. As none of the VIFs exceeded the threshold of 10 (54), multicollinearity among the independent variables was not considered problematic (**Supplementary Table 3**).

### 3.3 Latent profile analysis

**Figure 2:**
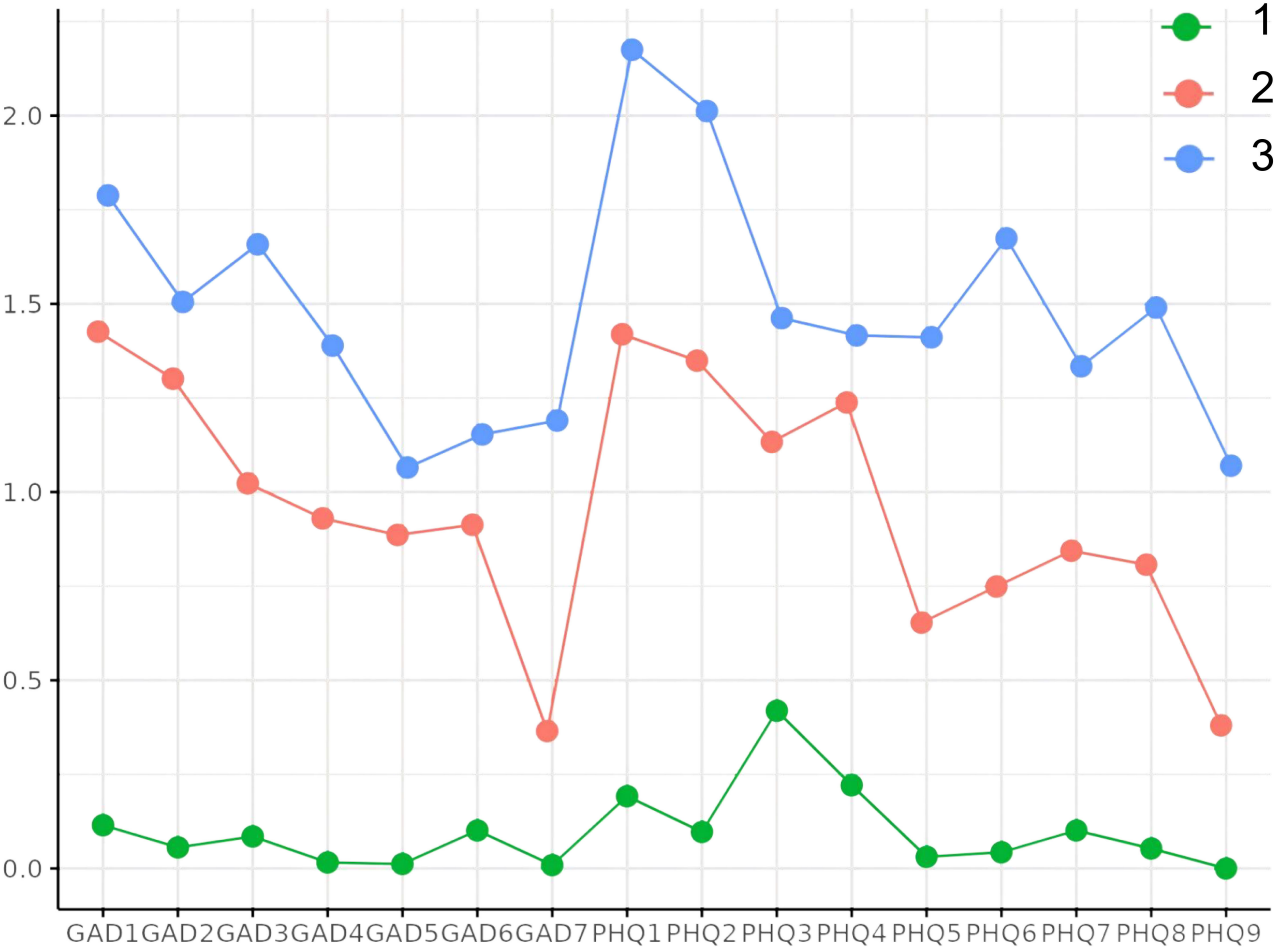
Each item scores of the PHQ-9 and GAD-7 for the three-class model’s profile plot.

LPA was performed using the PHQ-9 and GAD-7 scores as indicator variables. Models ranging from one to five classes were evaluated (**Table 2**). The BLRT yielded significant *P* for all models, and entropy values were above 0.9 for the two to five class solutions, indicating good classification precision. AIC, BIC and aBIC values decreased with each additional class. Although the four-class model exhibited an acceptable fit, it did not meet the minimum sample size requirement per class (n > 50) for model stability (55). Therefore, the three-class model was chosen as the optimal solution. The three latent classes were as follows: no depression-anxiety (Class 1: 67.93%), high-risk depression-anxiety (Class 2: 21.12%) and major depression-anxiety (Class 3: 10.95%). The mean PHQ-9 and GAD-7 scores for each class are presented in **Figure 2**. Item-level comparisons across classes are shown in **Table 3**. One-way ANOVA revealed significant differences in all item scores (*P* < 0.001). *Post hoc* Bonferroni tests indicated that Class 1 significantly differed from both Classes 2 and 3 across all items, while differences between Classes 2 and 3 were significant only for selected items.

**Table 2:** Fit indices for one-to-five-profile models of latent profile analysis.

Profile	AIC	BIC	aBIC	Entropy	BLRT *P*	Proportion (%)
1	17759.72	17894.71	17793.14	1.000	-	-
2	13513.37	13720.08	13564.55	0.987	0.002	0.30/0.70
3	12998.01	13276.44	13066.95	0.976	0.002	0.21/0.68/0.11
4	12688.10	13038.25	12774.80	0.977	0.002	0.07/0.67/0.12/0.14
5	12377.68	12799.54	12482.13	0.970	0.002	0.08/0.64/0.09/0.08/0.11

AIC, Akaike Information Criterion; BIC, Bayesian Information Criterion; aBIC, Sample Size Adjusted Bayesian Information Criterion; BLRT, Bootstrapped Likelihood Ratio Test.

**Table 3:** Mean differences in each item among latent profiles.

Items	CLASS 1 (M ± SD)	CLASS 2 (M ± SD)	CLASS 3 (M ± SD)	H
PHQ-9_1	0.19 (0.02)^ab^	1.42 (0.09)^c^	2.16 (0.10)	284.78***
PHQ-9_2	0.10 (0.02)^ab^	1.34 (0.08)^c^	2.02 (0.09)	349.70***
PHQ-9_3	0.42 (0.04)^ab^	1.13 (0.09)	1.47 (0.13)	105.56***
PHQ-9_4	0.22 (0.02)^ab^	1.24 (0.08)	1.42 (0.12)	199.64***
PHQ-9_5	0.03 (0.01)^ab^	0.66 (0.08)^c^	1.40 (0.12)	253.70***
PHQ-9_6	0.04 (0.01)^ab^	0.74 (0.07)^c^	1.69 (0.10)	309.92***
PHQ-9_7	0.10 (0.02)^ab^	0.85 (0.08)^c^	1.33 (0.12)	203.54***
PHQ-9_8	0.05 (0.01)^ab^	0.81 (0.09)^c^	1.47 (0.14)	227.21***
PHQ-9_9	0.00 (0.00)^ab^	0.38 (0.05)^c^	1.07 (0.11)	248.02***
PHQ-9	1.15 (0.09)^ab^	8.56 (0.27)^c^	14.04 (0.51)	341.70***
GAD-7_1	0.11 (0.02)^ab^	1.42 (0.08)^c^	1.80 (0.10)	326.37***
GAD-7_2	0.05 (0.01)^ab^	1.30 (0.08)	1.51 (0.11)	343.04***
GAD-7_3	0.08 (0.02)^ab^	1.02 (0.07)^c^	1.67 (0.10)	315.21***
GAD-7_4	0.01 (0.01)^ab^	0.93 (0.08)^c^	1.38 (0.12)	310.42***
GAD-7_5	0.01 (0.01)^ab^	0.89 (0.08)	1.07 (0.11)	278.50***
GAD-7_6	0.10 (0.02)^ab^	0.92 (0.08)	1.15 (0.11)	195.75***
GAD-7_7	0.01 (0.01)^ab^	0.36 (0.06)^c^	1.20 (0.11)	250.14***
GAD-7	0.38 (0.04)^ab^	6.83 (0.27)^c^	9.78 (0.38)	365.54***

a was statistical difference between class 1 and class 2; b was statistical difference between class 1 and class 3; c was statistical difference between class 2 and class 3; *Post-hoc* tests are based on the Bonferroni method.

Class 1, no depression-anxiety; Class 2, high-risk depression-anxiety; Class 3, major depression-anxiety; ***P < 0.001. PHQ-9: The Patient Health Questionnaire-9; GAD-7: The Generalized Anxiety Disorder-7.

### 3.4 Univariate analysis of depression-anxiety profiles in AIS patients

Univariate analysis identified several factors significantly associated with latent depression-anxiety profiles (*P* < 0.1), including: personal traits: hypertension、WST、age、PNI、SII、NLR、PLR; behavioral characteristics: BI and NIHSS; interpersonal network: SSRS; work/living conditions: occupation and CD-RISC-10 (**Table 1**).

### 3.5 Firth’s logistic regression of predictors of depression-anxiety profiles

To further explore factors associated with latent class membership, Firth’s logistic regression was performed. Variables with *P* < 0.1 in the univariate analysis were included in the model, with no depression-anxiety class serving as the reference category. For high-risk depression-anxiety class, significant predictors included: occupation (OR = 0.61, 95% CI [0.40–0.93]), hypertension (OR = 2.37, 95% CI [1.29–4.35]), NIHSS (OR = 1.60, 95% CI [1.06–2.42]) and BI (OR = 1.67, 95% CI [1.27–2.19]). For major depression-anxiety class, significant predictors were: NIHSS (OR = 2.33, 95% CI [1.42–3.85]), BI (OR = 2.65, 95% CI [1.62–4.35]) and CD-RISC-10 (OR = 0.92, 95% CI [0.87–0.98]). Full regression results are summarized in **Table 4**.

**Table 4:** Results of firth logistic regression.

Items	Class 2	*P*	Class 3	*P*
	OR (95% CI)		OR (95% CI)	
Age	0.99 (0.97 to 1.02)	0.485	1.01 (0.98 to 1.05)	0.473
WST	1.19 (0.95 to 1.48)	0.127	1.23 (0.93 to 1.63)	0.149
NIHSS	1.60 (1.06 to 2.42)	0.026	2.33 (1.42 to 3.85)	<0.001
Hypertension	2.37 (1.29 to 4.35)	0.005	2.32 (0.99 to 5.43)	0.053
PNI	0.97 (0.94 to 1.01)	0.198	1.01 (1.00 to 1.03)	0.141
SII	1.00 (0.99 to 1.00)	0.999	1.00 (0.99 to 1.00)	0.829
NLR	1.07 (0.89 to 1.29)	0.488	1.01 (0.78 to 1.29)	0.968
PLR	1.00 (0.99 to 1.00)	0.594	1.01 (1.00 to 1.01)	0.053
BI	1.67 (1.27 to 2.19)	<0.001	2.65 (1.62 to 4.35)	<0.001
CD-RISC-10	0.98 (0.94 to 1.02)	0.360	0.92 (0.87 to 0.98)	0.005
SSRS	1.00 (0.97 to 1.04)	0.777	1.00 (0.97 to 1.05)	0.828
Occupation	0.61 (0.40 to 0.93)	0.022	0.73 (0.41 to 1.33)	0.309

OR, Odds ratio; CI, Confidence interval; Class 2 = high-risk depression-anxiety; Class 3 = major depression-anxiety; WST: Water Swallowing Test; NIHSS: The National Institutes of Health Stroke Scale; PNI: Prognostic Nutritional Index; SII: Systemic Immune-inflammation Index; NLR: Neutrophil-to-Lymphocyte Ratio; PLR: Platelet-to-Lymphocyte Ratio; BI: Barthel Index; CD-RISC-10: the 10-item Connor-Davidson Resilience Scale; SSRS: Social Support Rating Scale.

## 4 Discussion

### 4.1 Latent profiles of depression and anxiety

In this study, LPA identified three distinct subgroups of psychological status among patients with AIS, highlighting substantial heterogeneity in co-occurring depression and anxiety symptoms. This person-centered analytic approach provides a more nuanced understanding of post-stroke psychological responses by classifying individuals based on symptom patterns, rather than relying on aggregated mean scores. Three latent classes were identified based on item response means: “no depression-anxiety” (Class 1: 67.93%), “high-risk depression-anxiety” (Class 2: 21.12%) and “major depression-anxiety” (Class 3: 10.95%). Class 1 demonstrated consistently low scores across all variables, indicating better overall psychological health. Class 2 is characterized by scores that fall between those of Class 1 and Class 3. Prior research has established a cutoff of 5 or higher on the PHQ-9 and GAD-7 as indicative of mild depression and anxiety, respectively (56, 57). In addition, other studies have identified thresholds for severe symptoms, defining major depression as a PHQ-9 score of 10 or above (58) and severe anxiety as a GAD-7 score of 8 or above (59). Given the mean PHQ-9 and GAD-7 scores in Class 2 (M_PHQ-9 = 8.56; M_GAD-7 = 6.83), this group comprises individuals exhibiting elevated levels of depressive and anxiety symptoms—levels that exceed the thresholds typically indicative of mild symptomatology and approach the severity observed in major depressive and anxiety disorders. These individuals are at increased risk of developing clinically significant psychological distress, justifying their classification as a distinct profile. Patients in Class 2, while not meeting diagnostic thresholds, occupy a vulnerable “borderline” state that may progress to more severe psychopathology if left unaddressed (58, 60). In contrast, Class 3 had significantly higher scores across all items, reflecting the presence of severe psychological symptoms. Notably, two items consistently yielded the highest scores in both Class 2 and Class 3: GAD-7_1 (“Feeling nervous, anxious, or on edge”) and PHQ-9_1 (“Little interest or pleasure in doing things”). These findings suggest that anhedonia and nervousness may represent core, early-emerging symptoms of post-stroke psychological distress. Future screening instruments should prioritize these sentinel items to facilitate early identification of at-risk individuals. Importantly, previous evidence indicates that screening alone is insufficient to improve patient outcomes (20). These findings highlight the critical need for interdisciplinary collaboration, integrating systematic detection with targeted psychological and rehabilitative interventions.

### 4.2 Determinants of psychological distress profiles

As a fundamental element of the HEM, individual characteristics play a pivotal role in shaping post-stroke psychological outcomes, with particular emphasis on hypertension and stroke severity. Hypertension, a well-established vascular risk factor (61), was independently associated with a 2.37-fold increased risk of comorbid PSD and PSA. A possible mechanism for this involves sympathetic nervous system overactivation in hypertensive individuals (62), leading to elevated catecholamine levels, which contribute to heightened arousal, anxiety and emotional dysregulation. Stroke severity, measured by NIHSS, was also a significant predictor of depression-anxiety profiles (Class 2: OR = 1.60; Class 3: OR = 2.33). More severe neurological impairments exacerbate emotional distress through various pathways, including physical disability, impaired communication and reduced autonomy, which hinder engagement in meaningful activities and increase the likelihood of social isolation (63–65).

In terms of behavioral characteristics, deficits in activities of daily living, as measured by the BI, were strongly associated with increased depression and anxiety risk (Class 2: OR = 1.67; Class 3: OR = 2.65). Reduced functional independence may undermine self-esteem and contribute to feelings of helplessness and identity loss (66). Moreover, the inability to participate in familial and social life may erode social support and contribute to psychological morbidity (67). Stroke survivors frequently encounter not only physical disabilities but also emotional instability and the redefinition of their social roles. These challenges play a critical role in escalating psychological distress. Resilience, defined as the ability to recover and adapt when confronted with adversity, emerged as an important protective factor for mental health (68). In this study, resilience was significantly linked to latent subgroup classification at three months post-stroke, serving as a buffer against psychological distress. This finding aligns with evidence from oncology research, where lower resilience is associated with a rapid escalation of negative emotional states, including depression and anxiety (69). Although many longitudinal studies have neglected the perspective of comorbidity, one investigation demonstrated that resilience independently predicts PSD across multiple time points within six months, reinforcing its role as a critical protective factor (70). Individuals with higher resilience tend to adopt more constructive perspectives when facing illness, which enhances engagement in rehabilitation and leads to better health outcomes (71–73). Therefore, early interventions designed to enhance resilience-related skills may substantially improve patients’ self-efficacy while concurrently reducing psychological distress, anxiety and depressive symptoms (74).

Regarding work and living conditions, the study suggests that engagement in occupational activities, including agricultural work, serves as a protective factor against both depression and anxiety in stroke survivors. Occupation plays a dual role, not only providing financial support but also forming a core element of personal identity, social integration and existential purpose (75). Regular involvement in work-related tasks enhances autonomy, perceived self-efficacy and a sense of accomplishment, which contributes to psychological well-being and improves overall quality of life (76, 77).

Turning to Interpersonal Networks, although univariate analysis identified a significant association between social support and psychological profiles, this effect was not sustained in the multivariable model. This may indicate that social support exerts indirect effects on emotional outcomes, possibly mediated through functional status or resilience (78). Alternatively, the impact of social support may be more significant during later stages of recovery than during the acute post-stroke period (79). Future studies employing longitudinal structural equation modeling could offer deeper insights into the mediating effects and temporal dynamics of social support on mental health.

### 4.3 Limitations and implications

Several limitations should be acknowledged in this study. First, the outcome measures were assessed at a single time point, three months post-AIS, which restricts our ability to examine the long-term trajectory of psychological profiles in AIS patients. Future research will address this limitation by examining the long-term trajectories of these psychological features. Second, the use of telephone follow-up to collect outcome data introduces potential reporting biases, which may affect the accuracy of the results. Thirdly, while the HEM provided a robust theoretical framework for identifying potential influencing factors, certain significant determinants were not incorporated into this study. Specifically, environmental, biological and psychosocial factors may have contributed to the observed associations. Future research should adopt a longitudinal design to track psychological outcomes over a longer period and utilize more objective data collection methods for follow-up assessments. Incorporating neuroimaging and other objective biomarkers, alongside the use of mediation analysis, would help clarify the underlying mechanisms. Furthermore, the potential mediating role of social support in the development of concurrent PSD and PSA warrants focused investigation.

### 4.4 Conclusions

This study identified three latent psychological profiles among AIS survivors: no depression-anxiety, high-risk depression-anxiety and major depression-anxiety, with clear inter-individual heterogeneity. Five key factors including occupation, hypertension, NIHSS, BI and resilience were significantly associated with profiles. Our findings support the integration of early psychological screening protocols into routine AIS care, particularly for individuals at high risk based on clinical and sociodemographic characteristics. Interventions designed to enhance resilience and facilitate occupational re-engagement should be prioritized to mitigate psychiatric morbidity, improve functional outcomes and promote holistic recovery in stroke patients.

## Data Availability

The raw data supporting the conclusions of this article will be made available by the authors, without undue reservation.
